# Acute and chronic effects of resistance training on skeletal muscle markers of mitochondrial remodeling in older adults

**DOI:** 10.14814/phy2.14526

**Published:** 2020-08-03

**Authors:** Paulo H.C. Mesquita, Donald A. Lamb, Hailey A. Parry, Johnathon H. Moore, Morgan A. Smith, Christopher G. Vann, Shelby C. Osburn, Carlton D. Fox, Bradley A. Ruple, Kevin W. Huggins, Andrew D. Fruge, Kaelin C. Young, Andreas N. Kavazis, Michael D. Roberts

**Affiliations:** ^1^ School of Kinesiology Auburn University Auburn AL USA; ^2^ Department of Nutrition, Dietetics and Hospitality Management Auburn University Auburn AL USA; ^3^ Department of Cell Biology and Physiology Edward Via College of Osteopathic Medicine Auburn AL USA

**Keywords:** aging, mitochondrial dynamics, mitochondrial function

## Abstract

We investigated the acute and chronic effects of resistance training (RT) on skeletal muscle markers of mitochondrial content and remodeling in older, untrained adults. Sixteen participants (*n* = 6 males, *n* = 10 females; age = 59 ± 4 years) completed 10 weeks of full‐body RT (2 day/week). Muscle biopsies from the vastus lateralis were obtained prior to RT (Pre), 24 hr following the first training session (Acute), and 72 hr following the last training session (Chronic). Protein levels of mitochondrial electron transport chain complexes I–V (+39 to +180%, *p* ≤ .020) and markers of mitochondrial fusion Mfn1 (+90%, *p* = .003), Mfn2 (+110%, *p* < .001), and Opa1 (+261%, *p* = .004) increased following chronic RT. Drp1 protein levels also increased (+134%, *p* = .038), while Fis1 protein levels did not significantly change (−5%, *p* = .584) following chronic RT. Interestingly, protein markers of mitochondrial biogenesis (i.e., PGC‐1α, TFAM, and NRF1) or mitophagy (i.e., Pink1 and Parkin) were not significantly altered (*p* > .050) after 10 weeks of RT. In summary, chronic RT promoted increases in content of electron transport chain proteins (i.e., increased protein levels of all five OXPHOS complexes) and increase in the levels of proteins related to mitochondrial dynamics (i.e., increase in fusion protein markers) in skeletal muscle of older adults. These results suggest that chronic RT could be a useful strategy to increase mitochondrial protein content in older individuals.

## Graphical abstract

The study by Mesquita et al. confirms that 10 weeks of resistance training increases markers of mitochondrial density as well as affects markers of mitochondrial dynamics.

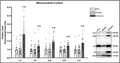

## 1 INTRODUCTION

Aging is a process characterized by a progressive decline in skeletal muscle health, which is associated with decreased quality of life and increased mortality in the elderly population (Berger & Doherty, 2010; Visser & Schaap, 2011). Besides decreases in muscle mass and strength (Berger & Doherty, 2010), termed sarcopenia, an age‐related reduction in skeletal muscle oxidative capacity (Short et al., 2005) and in overall cardiorespiratory fitness (Gonzalez‐Freire et al., 2018) has also been reported. However, researchers have postulated that the decreased physical activity and fitness commonly reported for older adults, and not aging per se, might be the cause of a reduced skeletal muscle mitochondrial function (Coen, Musci, Hinkley, & Miller, 2019). Although the causes of sarcopenia and reduced skeletal muscle function are multifactorial, mitochondria have been considered to play an important role in these processes (Jang, Blum, Liu, & Finkel, 2018; Marzetti et al., 2014; Sun, Youle, & Finkel, 2016).

In skeletal muscle, mitochondria exist as a highly dynamic network that responds to the energy demands of the cell. Mitochondria undergo constant remodeling through the generation of new mitochondria (biogenesis), joining (fusion), splitting (fission), and degradation of dysfunctional portions (mitophagy) (Youle & Bliek, 2012). Adequate remodeling processes are essential for maintaining functional mitochondria (Tilokani, Nagashima, Paupe, & Prudent, 2018; Youle & Bliek, 2012). Fusion of the outer mitochondrial membrane is mediated by the Mitofusins 1 and 2 (Mfn1 and Mfn2), while Optic Atrophy 1 (Opa1) mediates the fusion of the inner mitochondrial membrane (Pernas & Scorrano, 2016). The fission process is carried out by Dynamin related protein 1 (Drp1), which is recruited from the cytosol by Fission protein 1 (Fis1) (Yoon, Krueger, Oswald, & McNiven, 2003). Lastly, mitophagy occurs mainly through the PTEN‐induced putative kinase 1 (Pink1)/Parkin pathway (Youle & Narendra, 2011). Pink1 accumulates in the outer mitochondrial membrane of damaged mitochondria and recruits Parkin, which promotes their elimination (Youle & Narendra, 2011).

Exercise is considered an important strategy to prevent the decline in muscle function during aging and to improve or maintain mitochondrial function. Resistance training (RT) is a well‐known method to increase muscle mass and strength at any age (Folland & Williams, 2007), while endurance training promotes mitochondrial adaptations (Holloszy, 1967; Konopka, Suer, Wolff, & Harber, 2014). However, there is a growing body of literature investigating the mitochondrial adaptations to RT in both younger (Alvehus, Boman, Söderlund, Svensson, & Burén, 2014; Lim et al., 2019; Porter, Reidy, Bhattarai, Sidossis, & Rasmussen, 2015) and older (Flack et al., 2016; Manfredi et al., 2013; Miller et al., 2020; Zampieri et al., 2016) participants, with mixed results. As pointed out by Parry, Roberts, & Kavazis (2020), RT may impose a sufficient energetic stimulus to promote mitochondrial adaptations in older participants. Although there is a growing interest in the effects of RT on mitochondrial biogenesis in older participants, there is a paucity of research investigating its effects on markers of mitochondrial remodeling, which is essential for quantity and quality control of the mitochondrial network. Therefore, the aim of this study was to investigate the acute and chronic effects of RT on markers of mitochondrial content and remodeling in older, untrained individuals.

## 2 METHODS

### 2.1 Ethics approval

The current investigation was a secondary analysis of a study that aimed to explore the effects of RT with peanut protein supplementation on skeletal muscle hypertrophy in older untrained subjects (Lamb et al., 2020). Notably, participants either consumed ~72 g per day of a peanut supplement providing ~30 g protein (PBfit; Betterbody Foods, Lindon, UT, USA) or no supplement in an unblinded fashion (NCT04015479). The study was approved by the Institutional Review Board at Auburn University (Protocol # 19–249 MR 1907) and was carried out in compliance with the Declaration of Helsinki. Two‐way repeated measures analysis of variance (ANOVA) showed no effects of peanut protein supplementation on any of the variables investigated in this study (*p* > .05 for all variables). Therefore, the supplemented and nonsupplemented groups were combined for analysis. All participants were informed about the procedures and possible risks of the study and provided written consent prior to participation.

### 2.2 Participants

Sixteen older (*n* = 6 males, *n* = 10 females; age = 59 ± 4 years) participants without recent RT experience were recruited to participate in the study. Inclusion criteria required participants to be 55–80 years old, have a body mass index of less than 35, have not been participating in RT for more than 2 days/week, and to abstain from nutritional supplementation one month prior to enrollment. Participants also had to be free of overt cardio‐metabolic diseases (e.g., type II diabetes, severe hypertension, heart failure) or conditions that precluded the collection of a skeletal muscle biopsy. Notably, none of the participants had reported structured RT over the past year.

### 2.3 Study design

Participants visited the laboratory on three different occasions, during which a biopsy of the vastus lateralis (VL) was taken for later molecular analyses. All visits occurred at the same time of the day, and biopsies occurred in the morning hours following an overnight fast. Three participants, however, reported to the laboratory during evening hours following a ~4–5 hr fast. The first visit (Pre) occurred ~2–5 days prior to the first day of RT. Participants then started the RT program (details below) and reported to the lab for the second biopsy 24 hr following the first training session (Acute). The training program continued for 10 weeks and participants had the third biopsy taken 72 hr after the last training session (Chronic). Additional testing to evaluate the effects of the RT program on body composition, muscle thickness of the VL, and peak torque for right leg extensors were also performed during each visit and have been described and published previously (Lamb et al., 2020).

### 2.4 Resistance training program

A complete description of the RT program can be found in Lamb et al. (2020). Briefly, the training program was supervised, lasted 10 weeks in duration, and consisted of a whole‐body workout performed twice weekly. During each session, participants performed three sets of 10–12 repetitions of leg press, leg extensions, leg curls, barbell bench press, and cable pull downs, with at least 1 min of rest between sets. At the end of each set, the level of difficulty was rated by participants (0 = easy, 10 = hard). If the participant rated the set difficulty below 7, weight was modestly adjusted to increase resistance. If the participant rated the set difficulty at 10, or the participant was not able to complete the set because of difficulty, weight was removed. Participants were consistently instructed by staff members to be as truthful as possible when assessing the difficulty of sets. Our goal was to ensure participants gauged sets between a 7 and 9 rating.

### 2.5 Muscle biopsies

Muscle biopsies were taken before (Pre), 24 hr following the first training session (Acute), and 72 hr following the last training session (Chronic). A 5‐gauge needle was used to obtain biopsies of the right leg VL as previously described by our laboratory (Kephart et al., 2015). Following biopsies, tissue was rapidly teased of blood and connective tissue, flash frozen using liquid nitrogen, and subsequently stored at −80°C for future use.

### 2.6 Western blotting

Muscle samples were removed from −80°C and crushed on a liquid nitrogen‐cooled ceramic mortar using a ceramic pestle. Approximately 20 mg of tissue was placed in 200 μl of lysis buffer (25 mM Tris, pH 7.2, 0.5% Triton X‐100, 1x protease inhibitors) and homogenized using tight‐fitting plastic pestles. Samples were centrifuged at 1,500 *g* for 10 min at 4°C. Supernatants were then collected and used to determine protein concentrations using a commercially available bicinchoninic acid kit (Thermo Fisher Scientific; Walthan, MA, USA). Afterwards, supernatants were prepared for Western blotting using 4x Laemmli buffer and distilled water (diH2O).

Samples (12 μl) were pipetted onto gradient sodium dodecyl sulfate‐polyacrylamide gels (4%–15% Criterion TGX Stain‐free gels; Bio‐Rad Laboratories; Hercules, CA, USA), and proteins were separated by electrophoresis (200 V for approximately 45 min). After electrophoresis, proteins were transferred to preactivatedpolyvinylidene difluoride membranes (Bio‐Rad Laboratories) for 2 hr at 200 mA. Gels were then Ponceau stained for 3 min, washed with diH2O for 30 s, quickly dried, and digitally imaged with a gel documentation system (UVP, LLC, Upland, CA, USA). Following Ponceau imaging, membranes were reactivated in methanol, blocked with nonfat milk for 1 hr (5% w/v diluted in Tri‐buffered saline with 0.1% Tween 20, or TBST (Tris‐buffered saline with Tween 20)), washed three times in TBST only (Berg et al., 2020), and incubated for 1 hr with primary antibodies (1:2000 v/v dilution in TBST with 5% BSA). Primary antibodies were used to detect: Total OXPHOS Human Cocktail (Abcam Cat# ab110411, RRID:AB_2756818), COX IV (Cell Signaling Technology Cat# 4850, RRID:AB_2085424), PGC‐1α (GeneTex Cat# GTX37356, RRID:AB_11175466), NRF1 (GeneTex Cat# GTX103179, RRID:AB_11168915), TFAM (Abnova Corporation Cat# H00007019‐D01P, RRID:AB_1715621), Mfn1 (Cell Signaling Technology Cat# 14739, RRID:AB_2744531), Mfn2 (BioVision Cat# 3882‐100, RRID:AB_2142625), Opa1 (Cell Signaling Technology Cat# 67589, RRID:AB_2799728), Fis1 (Abcam Cat# ab71498, RRID:AB_1271360), Drp1 (Novus Cat# NB110‐55288SS, RRID:AB_921147), Pink1 (Cell Signaling Technology Cat# 6946, RRID:AB_11179069), and Parkin (Cell Signaling Technology Cat# 2132, RRID:AB_10693040). Due to the protocol utilized, we were unable to detect complex IV in Total OXPHOS Human Cocktail, which has been previously reported (Herbst et al., 2014; Miotto, McGlory, Holloway, Phillips, & Holloway, 2018). Therefore, we interrogated COX IV as an individual target. Validation of the antibodies used has been previously reported (Balan et al., 2019; Campbell, To, & Spector, 2019; Liu, Peyton, & Durante, 2013; Ordureau et al., 2018; Parry et al., 2019; Pillon et al., 2020; Radde et al., 2016; Tarpey et al., 2019; Yao et al., 2019; Zhang et al., 2017; Zhong et al., 2019). Following primary antibody incubations, membranes were washed three times in TBST only for 5 min, and incubated for 1 hr with horseradish peroxidase‐conjugated anti‐rabbit IgG (Cell Signaling Technology Cat# 7074, RRID:AB_2099233) or anti‐mouse IgG (Cell Signaling Technology Cat# 7076, RRID:AB_330924). Membranes were then washed in TBST only (3x5 min), developed using chemiluminescent substrate (Millipore; Burlington, MA, USA), and digitally imaged in a gel documentation system (UVP, LLC, Upland, CA, USA). Raw target band densities were obtained using imaging software ImageJ (NIH, Bethesda, MD, USA), and the values were normalized to Ponceau staining. These values were then divided by the mean of baseline values (Pre) to obtain fold‐difference values.

### 2.7 Statistics

All statistical analyses were performed using SPSS v21.0 (IBM Corp, Armonk, NY, USA). Data are expressed as mean ± standard deviation (*SD*). Repeated measures ANOVAs were performed to examine the effects of RT on individual targets. The sphericity assumption on all dependent variables was tested using the Mauchly's test, and the Greenhouse–Geisser correction was used when the sphericity assumption was violated. Post hoc Bonferroni tests were used when appropriate. Statistical significance was established at *p* < .050.

## 3 RESULTS

### 3.1 Participant characteristics and training adaptations

Although participant characteristics and certain training adaptations were reported in Lamb et al. (2020), they are listed here for convenience to the reader. The participant cohort was made up of *n* = 6 males and 10 females. The average age of participants prior to training was 59 ± 4 years of age. Participants had a body mass index of 31.7 ± 5.6 kg/m^2^, a fat‐free mass index (FFMi; DXA FFM in kg divided by height in m^2^) of 18.0 ± 2.9 kg/m^2^, and a body fat percentage of 39.3 ± 6.3%; the latter two variables being determined by dual energy X‐ray absorptiometry (DXA).

Regarding training adaptations, the participants experienced an increase in DXA FFM (±1.0 ± 1.9 kg), albeit this trended towards significance (*p* = .061). However, VL muscle thickness (assessed using an ultrasound) increased from 1.88 ± 0.45 cm to 2.02 ± 0.37 cm with training, and knee extensor peak torque at 60°/s increased from 115 ± 43 to 127 ± 40 N m with training. Both of these increases were significant (*p* < .05).

### 3.2 Mitochondrial content

**FIGURE 1:**
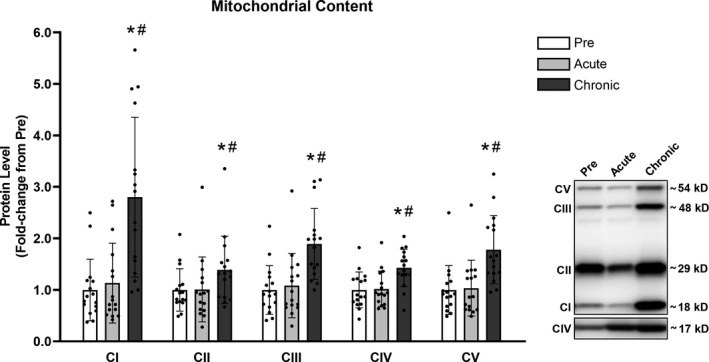
Chronic RT significantly increased protein levels of complexes I to V. Legend: CI to V, Complex I to V; Pre, protein levels at baseline; Acute, protein levels at 24 hr after the first training session; Chronic, protein levels at 72 hr after the last training session; *, significantly different from Pre (*p* < .050); #, significantly different from Acute (*p* < .050). Data are presented as means ± *SD*, *n* = 16 (6 men, 10 women)

Acute RT did not significantly affect the skeletal muscle protein levels of any of the electron transport chain complexes analyzed (CI: +13% [*p* = 1.000], CII: +1% [*p* = 1.000], CIII: +8% [*p* = 1.000], CIV: +2% [*p* = 1.000], CV: +3% [*p* = 1.000]). However, at the end of 10 weeks of training, all five complexes had increased protein levels compared to baseline values (CI: +180% [*p* < .001], CII: +39% [*p* = .020], CIII: +89% [*p* < .001], CIV: +43% [*p* < .001], CV: +78% [*p* < .001]) (Figure 1).

### 3.3 Mitochondrial biogenesis

**FIGURE 2:**
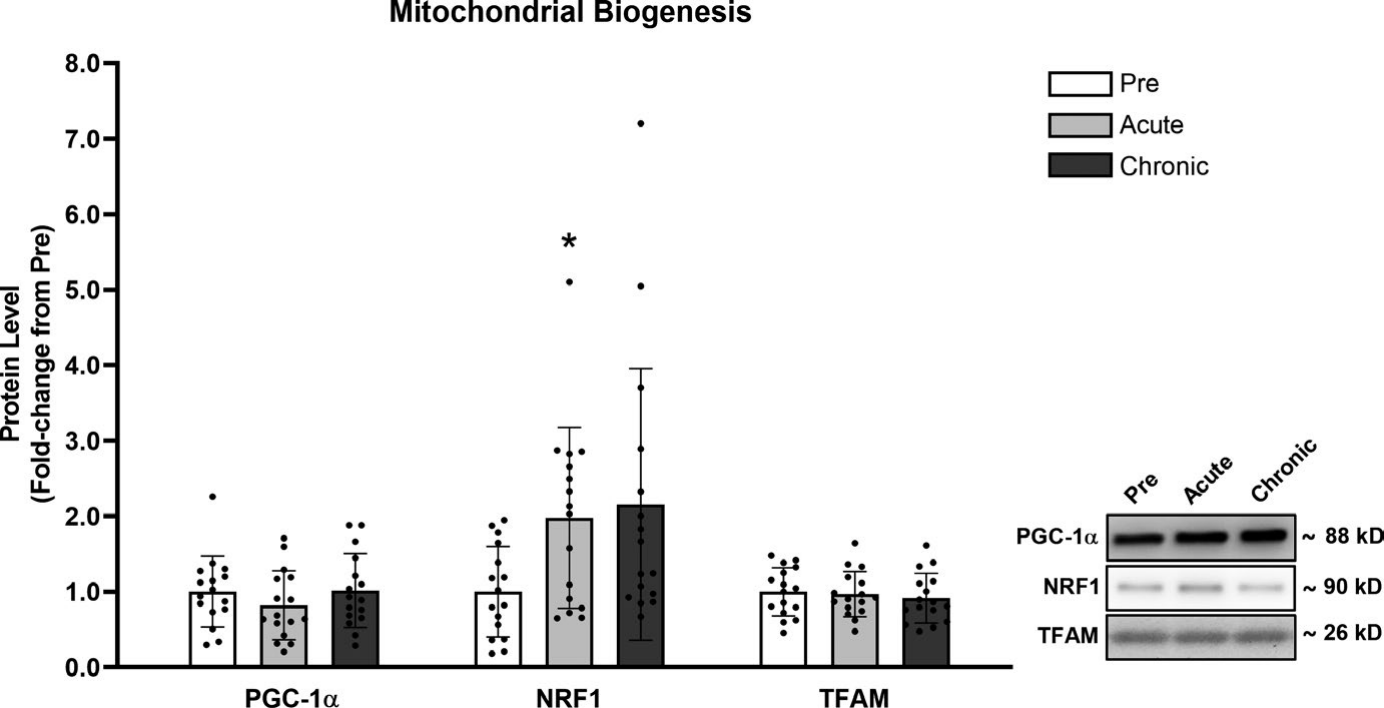
RT acutely increased NRF1 but did not alter PGC‐1α or TFAM protein levels. Legend: Pre, protein levels at baseline; Acute, protein levels at 24 hr after the first training session; Chronic, protein levels at 72 hr after the last training session; *, significantly different from Pre (*p* < .050). Data are presented as means ± *SD*, *n* = 16 (6 men, 10 women)

No significant differences (*p* > .050) were detected for skeletal muscle PGC‐1α or TFAM protein levels following acute or chronic RT. NRF1 protein levels were elevated following acute RT (+98%, *p* = .019) and albeit not statistically significant, trended to increase following chronic RT (+116%, *p* = .082) (Figure 2).

### 3.4 Mitochondrial fusion

**FIGURE 3:**
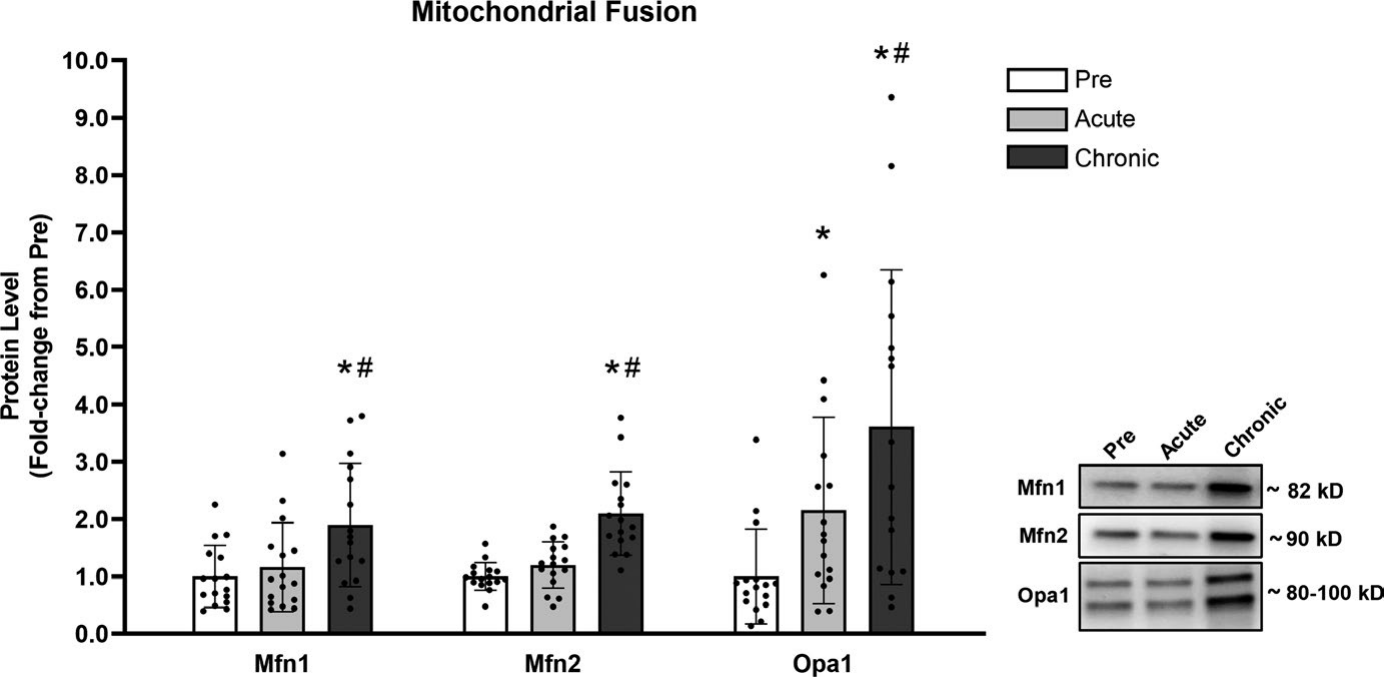
Chronic RT significantly increased markers of mitochondrial fusion. Legend: Pre, protein levels at baseline; Acute, protein levels at 24 hr after the first training session; Chronic, proteins level at 72 hr after the last training session; *, significantly different from Pre (*p* < .050); #, significantly different from Acute (*p* < .050). Data are presented as means ± *SD*, *n* = 16 (6 men, 10 women)

Mfn1 and Mfn2 protein levels of skeletal muscle did not significantly change following acute RT (Mfn1: +16% (*p* = .430), Mfn2: +20%, (*p* = .207)) but increased following chronic RT (Mfn1: +90% (*p* = .003), Mfn2: +110% (*p* < .001)). Opa1 protein levels, on the other hand, increased following acute (+115%, *p* = .011) and chronic RT (+261%, *p* = .004) (Figure 3).

### 3.5 Mitochondrial fission

**FIGURE 4:**
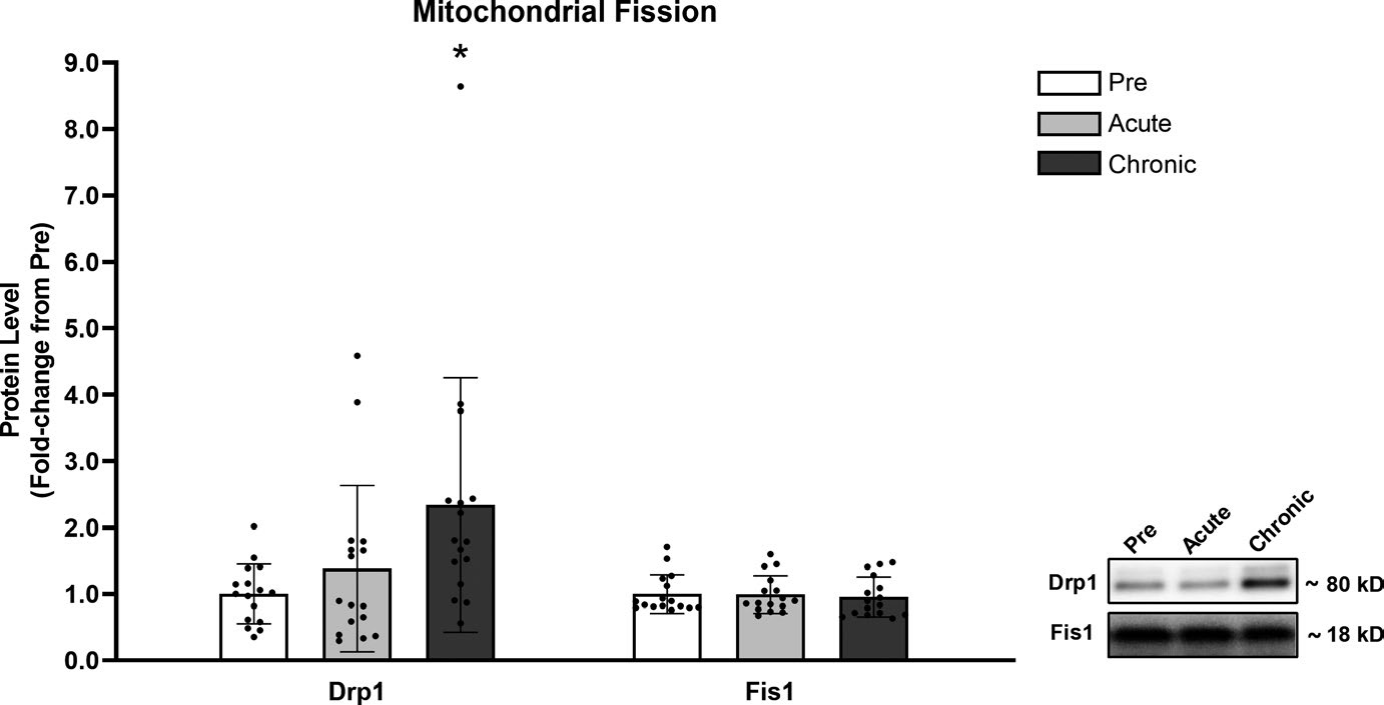
Drp1 protein levels significantly increased following chronic RT, while Fis1 remained unchanged. Legend: Pre, protein levels at baseline; Acute, protein levels at 24 hr after the first training session; Chronic, protein levels at 72 hr after the last training session; *, significantly different from Pre (*p* < .050). Data are presented as means ± *SD*, *n* = 16 (6 men, 10 women)

Drp1 protein levels did not significantly change following acute RT (+38%, *p* = .443), but increased following chronic RT (+134%, *p* = .038). Fis1 protein levels did not present any significant changes in response to acute (−1%, *p* = .584) or chronic (−5%, *p* = .584) RT (Figure 4).

### 3.6 Mitophagy

**FIGURE 5:**
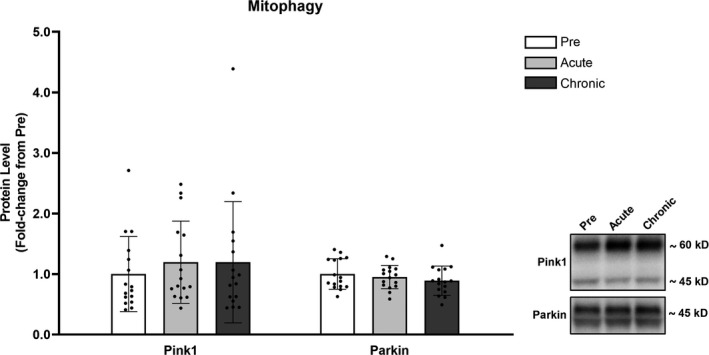
Mitophagy was not significantly altered in response to RT. Legend: Pre, protein levels at baseline; Acute, protein levels at 24 hr after the first training session; Chronic, protein levels at 72 hr after the last training session. Data are presented as means ± *SD*, *n* = 16 (6 men, 10 women)

No significant differences (*p* > .050) were detected for Pink1 or Parkin protein levels following acute or chronic RT (Figure 5).

## 4 DISCUSSION

Resistance training has been well recognized as an effective method to increase muscle hypertrophy and strength (Folland & Williams, 2007). However, whether or not RT induces mitochondrial adaptations remains equivocal and poorly investigated, especially in older individuals (Parry et al., 2020). We investigated the acute and chronic effects of RT on markers of mitochondrial content and remodeling in older individuals. Our findings demonstrated that 10 weeks of RT led to increases in mitochondrial OXPHOS protein content and dynamics, although only mild acute stimulus for mitochondrial biogenesis was detected.

A previous study conducted by our group with the same cohort investigated herein showed that the RT protocol led to significantly increased strength, localized hypertrophy, and several markers of skeletal muscle mitochondrial metabolism (Lamb et al., 2020). In addition, we previously reported that a robust increase in citrate synthase activity was observed, suggesting increased mitochondrial content in response to RT. Therefore, the increased protein levels of complexes I to V observed in this study is in agreement with the previous findings. Our results are also in agreement with other studies that found increased mitochondrial content in older subjects in response to RT (Jubrias, Esselman, Price, Cress, and Conley, 2001; Manfredi et al., 2013; Robinson et al., 2017). Robinson et al. (2017), for example, showed via proteomics that 12 weeks of RT increased mitochondrial protein abundance in older participants, albeit there was no improvement in mitochondrial function. However, it is important to note that no significant changes in mitochondrial markers have also been reported with RT in younger (Porter et al., (2015)) and older populations (Flack et al., 2016; Irving et al., 2019; Parise, Brose, & Tarnopolsky, 2005). A recent study conducted by Berger and Doherty (2010) showed a somewhat surprising decrease in mitochondrial function but no change in markers of mitochondrial content following 8 weeks of maximal strength training in older adults. While reasons for these equivocal findings are difficult to reconcile, we posit that this may be related to both the characteristics of the training protocol and to the fitness and physical activity levels of the participants. Training protocols that elicit greater metabolic demands seem to be more beneficial to mitochondrial adaptations (Lim et al., 2019). Furthermore, physical activity levels and fitness are possible confounding factors in the relationship between mitochondria and the aging process, in a way that a decline in physical activity, and not aging per se, is what causes a decrease in mitochondrial content or function (Coen et al., 2019). Therefore, RT might only be beneficial to individuals who are poorly conditioned prior to intervention, acting to restore a “healthy” mitochondrial status. The RT protocol adopted in this study was probably enough of a stimulus to elicit oxidative adaptations given that these participants were poorly conditioned prior to RT. In this regard, future studies are needed to determine how preexisting physical activity levels in older participants affect mitochondrial adaptations to RT.

Given the increased mitochondrial protein content in response to chronic RT, levels of proteins involved in the transcriptional control of mitochondrial biogenesis were also expected to be increased. Mitochondrial biogenesis requires a coordinated regulation of transcription of both nuclear and mitochondrial genes. Peroxisome proliferator‐activated receptor‐γ coactivator 1α (PGC‐1α) is considered to be a key regulator of mitochondrial biogenesis (Handschin & Spiegelman, 2006; Jornayvaz & Shulman, 2010). PGC‐1α interacts with and activates NRF1, increasing nuclear transcription of mitochondrial genes (Hood, 2009). In addition, PGC‐1α also controls the expression of the mitochondrial transcriptional factor TFAM, a key regulator of mitochondrial DNA expression (Wu et al., 1999). Despite the clear roles of PGC‐1α, NRF1, and TFAM on mitochondrial biogenesis, the only significant change detected in this study was the acute increase in NRF1 protein levels. Ogborn et al. (2015) investigated the response of PGC‐1α, NRF1, and TFAM mRNA levels following one bout of resistance exercise in older subjects. The authors observed no change in NRF1 mRNA, and initial increases in PGC‐1α and TFAM (at 3 and 24 hr postexercise, respectively) followed by a return to baseline levels 48 hr following exercise. Another study also reported that chronic RT did not alter PGC‐1α or TFAM mRNA levels in older participants (Flack et al., 2016). However, as highlighted by Robinson et al. (2017), adaptations at the transcriptional level do not necessarily imply proteomic adaptations. Thus, care should be taken when comparing the results of studies that used different measures. Irving et al. (2019) found an increase in the protein levels of PGC‐1α but no change in TFAM after 8 weeks of RT. Similar contradictory results showing increased mitochondrial content but no change in PGC‐1α, NRF1, or TFAM have been reported in young individuals in response to RT (Lim et al., 2019) and in older subjects in response to endurance training (Balan et al., 2019). Although there is no clear explanation for the overall lack of change in biogenesis markers despite the substantial increase in mitochondrial content found in this study, the contradictory results might be related to the timing of biopsy. Furthermore, researchers have recently reevaluated the role of PGC‐1α on mitochondrial biogenesis and showed that other proteins, such as PPARβ, may also be important in regulating mitochondrial biogenesis (Islam & Bonafiglia, 2019; Islam, Hood, & Gurd, 2020). Future studies should investigate how RT may be affecting other regulators of mitochondrial biogenesis in both younger and older individuals.

Besides mitochondrial biogenesis, the control of mitochondrial quantity and quality depends on the integration of coordinated events such as fusion, fission, and mitophagy. Studies have shown that proper mitochondrial dynamics are impaired during aging (Marzetti et al., 2014) and that exercise is an important strategy for counteracting such effects (Marzetti et al., 2014; Ziaaldini, Hosseini, & Fathi, 2017). In this study, RT did not acutely change any of the proteins related to mitochondrial dynamics. However, Mfn1, Mfn2, Opa1, and Drp1 were increased following 10 weeks of RT. The observed changes point to a scenario of increased mitochondrial fusion. Our results are in agreement with a study that investigated the effects of RT on mitochondrial dynamics in rats (Kitaoka, Ogasawara, Tamura, Fujita, & Hatta, 2015). The authors showed that chronic RT led to increased fusion but did not change fission. Four months of endurance training has also been shown to induce the same adaptations in older participants (Arribat et al., 2019). Lim et al. (2019), in turn, showed that lower‐load higher‐volume RT, believed to impose a greater metabolic demand, increased Fis1, Drp1, and Opa1 protein levels from biopsies obtained in young participants, but did not change Mfn2 protein levels. The differences in response between younger and older individuals might be related to an already unbalanced dynamics in favor of fission with aging (Carter, Chen, & Hood, 2019). This increased fission leads to a fragmented mitochondrial network, which in turn is linked to mitochondrial dysfunction (Marzetti et al., 2014; Zemirli, Morel, & Molino, 2018). Therefore, the increased fusion observed in this study could be a positive adaptation to counteract age‐related increased fission. Further, increased mitochondrial damage is reported with aging (Johnston, De, & Parise, 2008; Tarnopolsky, 2009) and fusion could have a role in mixing the contents of two mitochondria and possibly diluting damaged material. Thus, our data support the fact that RT positively affects mitochondrial fusion markers, and this adaptation may have led to an enhancement in mitochondrial function.

As previously stated, mitophagy is another essential process for mitochondrial quality control, as it removes dysfunctional portions of mitochondria. Mitophagy is believed to be impaired with aging, and an age‐related impairment in mitophagy could be linked to the accumulation of damaged mitochondria with aging (Jang et al., 2018). Exercise has been considered a strategy to increase mitophagy and therefore counteract such processes (Carter et al., 2019; Drake, Wilson, & Yan, 2019). Our results showed no change in Pink1 and Parkin protein levels in response to acute or chronic RT, which is not surprising considering that fission precedes mitophagy and a more robust increase in markers of mitochondrial fusion compared to fission was detected. Another study has shown that Parkin protein levels in rat skeletal muscle are not altered in response to RT (Kitaoka et al., 2015). Moreover, protein levels of Pink1 and Parkin have been shown to not be altered in older human participants following RT (Ogborn et al., 2015). On the other hand, Lim et al. (2019) reported increases in mitophagy protein markers in younger participants in response to RT, especially in the group performing lower‐load higher‐volume RT. It is possible that the discrepancy in the results is related to the different training protocols used and that a greater metabolic stress is needed to stimulate mitophagy. It is important to note that our results do not necessarily mean unaltered mitophagy. Pink1 and Parkin are subjected to phosphorylation events that impact their activity (Zhuang, Li, Chen, & Wang, 2016) and the levels of these phosphorylated proteins were not analyzed in this study. In addition, we cannot rule out the possibility that mitophagy was increased through Pink1/Parkin‐independent pathways. Notwithstanding, these preliminary data suggest that RT does not alter mitophagy markers in older individuals.

### 4.1 Experimental considerations

Limitations of this study include the fact that we did not have a control group of younger participants, and therefore, the changes observed herein might not be age‐specific. Furthermore, mitochondrial function can differ between males and females (Ferreira, 2018; Miotto et al., 2018), but our sample size did not enable such comparisons to be made in this study. Another limitation is that the acute responses to RT were determined at single time point (24 hr following the first training session). Therefore, our data regarding the acute responses must be interpreted with caution, as we may have missed important changes in the markers analyzed. In addition, even though changes in proteins related to mitochondrial content and dynamics were detected, measures of oxidative phosphorylation were not conducted due to tissue limitations. It is important to note, however, that studies have linked increased protein content to the observed increased mitochondrial function in response to RT before (Holloway et al., 2018; Porter et al., 2015). Even though such observations regarding markers of mitochondrial remodeling are scarce, studies have also demonstrated that markers of mitochondrial fusion (e.g., Mfn1 and Mfn2) can directly impact mitochondrial function (Bach et al., 2003; Chen, Chomyn, & Chan, 2005). As stated prior, mitochondrial dysfunction is intricately linked with muscle aging (Jang et al., 2018; Marzetti et al., 2014), and such measures could provide insightful information about the potential benefits of RT.

## 5 CONCLUSIONS

In conclusion, the results of this study showed that 10 weeks of RT increased mitochondrial protein content and markers of mitochondrial dynamics, although no changes in these markers were detected following the first training bout. The results suggest that the acute response may not be representative of the chronic effects of RT, and that repeated bouts are necessary to achieve mitochondrial benefits of RT in older populations. Critically, besides the known improvements in muscle mass and strength, RT could be a viable approach to improve the levels of proteins involved in oxidative phosphorylation and in mitochondrial dynamics.

## Disclosures

MDR and KCY perform contracted studies for nutritional supplement companies, but they nor do any of the other authors have financial or other conflicts of interest to report with regard to these data.

## AUTHORS CONTRIBUTIONS

PHCM, ADF, KCY, KWH, ANK, MDR conceived and designed the research; PHCM, DAL, JHM, MAS, CGV, SCO, CDF, BAR, ADF, KCY, ANK, and MDR performed experiments; PHCM, ANK, and MDR the analyzed data; PHCM, ANK, and MDR interpreted the results of experiments; PHCM prepared figures; PHCM drafted manuscript; PHCM, DAL, HAP, JHM, MAS, CGV, SCO, CDF, BAR, KWH, ADF, KCY, ANK, and MDR edited and revised the manuscript; PHCM, DAL, HAP, JHM, MAS, CGV, SCO, CDF, BAR, KWH, ADF, KCY, ANK, and the MDR approved final version of the manuscript.
